# Developing a predictive nomogram for mortality in patients with extrapulmonary acute respiratory distress syndrome: the prognostic value of serum soluble thrombomodulin, lung ultrasound score, and lactate

**DOI:** 10.3389/fphar.2024.1407825

**Published:** 2024-08-27

**Authors:** Yang Yang, Yue Wang, Guoguo Zhu, Siya Xu, Jie Liu, Zhongzhi Tang

**Affiliations:** ^1^ Department of Intensive Care Unit, Hefei BOE Hospital Co., Ltd., Hefei, Anhui, China; ^2^ Department of Science and Education, Hefei BOE Hospital Co., Ltd., Hefei, Anhui, China; ^3^ Department of Emergency, Central Theater General Hospital of the People’s Liberation Army of China, Wuhan, Hubei, China

**Keywords:** extrapulmonary acute respiratory distress syndrome, mortality, soluble thrombomodulin, lung ultrasound score, lactate, nomogram

## Abstract

**Objective:** This study aimed to elucidate the prognostic significance of serum soluble thrombomodulin (sTM), lung ultrasound score (LUS), and lactate levels in patients with extrapulmonary acute respiratory distress syndrome (ARDS), with the goal of refining mortality risk prediction in this cohort.

**Methods:** In a prospective cohort of 95 patients with extrapulmonary ARDS admitted to the intensive care unit, we investigated the primary endpoint of 28-day mortality. Utilizing Lasso-Cox regression analysis, we identified independent prognostic factors for mortality. A predictive nomogram was developed incorporating these factors, and its performance was validated through several statistical measures, including the consistency index, calibration plot, internal validation curve, decision curve analysis, interventions avoided analysis, receiver operating characteristic curve analysis, and Kaplan-Meier survival analysis. We further conducted a subgroup analysis to examine the impact of prone positioning on patient outcomes.

**Results:** The study identified baseline serum sTM, LUS, and lactate levels as independent predictors of 28-day mortality in extrapulmonary ARDS patients. The predictive nomogram demonstrated superior prognostic accuracy compared to the use of sTM, LUS, or lactate levels alone, and outperformed traditional prognostic tools such as the Acute Physiology and Chronic Health Evaluation II score and the partial pressure of arterial oxygen to fractional inspired oxygen ratio. The subgroup analysis did not show a significant impact of prone positioning on the predictive value of the identified biomarkers.

**Conclusion:** Our study results support the development and validation of a novel prognostic nomogram that integrates key clinical biomarkers and ultrasound imaging scores to predict mortality in patients with extrapulmonary ARDS. While our research is preliminary, further studies and validation are required.

## 1 Introduction

Acute respiratory distress syndrome (ARDS) is a clinical syndrome marked by an acute onset and refractory hypoxemia, primarily resulting from damage to capillary endothelial cells and alveolar epithelial cells (Suresh et al., 2000). ARDS poses a significant challenge in intensive care units (ICUs). Approximately 10.4% of ICU patients rapidly develop ARDS, with a mortality rate approaching 40% (Bellani et al., 2016), and an estimated 40% of ARDS cases may go undiagnosed (Laffey et al., 2016). Current therapeutic strategies for ARDS are limited, primarily relying on lung-protective ventilation (Peltan et al., 2023), fluid management (Ingelse et al., 2019), prone positioning (PP) (Al Hashim et al., 2023), and adjunctive anti-inflammatory and supportive therapies. While extracorporeal membrane oxygenation (ECMO) and lung transplantation are options for refractory ARDS (Cerier and Bharat, 2023; Majithia-Beet et al., 2023), they are costly and associated with high mortality rates. In recent years, stem cell therapy has shown promise for ARDS treatment, but it has not yet translated into effective clinical protocols (Lin et al., 2023). Consequently, early identification and proactive intervention for ARDS patients with poor prognosis are critical to improving survival rates.

ARDS is a highly heterogeneous syndrome, characterized by differences in etiology, pathology, pathophysiology, respiratory mechanics, and biomarkers (Viswan et al., 2020; Liao et al., 2023; Xu et al., 2023). Based on the underlying causes, ARDS can be categorized into pulmonary ARDS and extrapulmonary ARDS. Pulmonary ARDS typically results from direct lung injuries such as pneumonia or aspiration, whereas extrapulmonary ARDS is often caused by systemic diseases like pancreatitis, trauma, or sepsis (Bernard et al., 1994). The primary distinction between pulmonary and extrapulmonary ARDS lies in whether the injury originates from alveolar epithelial cells or vascular endothelial cells (Shaver and Bastarache, 2014). Studies have highlighted differences between these two types in terms of pathology (Negri et al., 2002; Menezes et al., 2005), respiratory mechanics (Gattinoni et al., 1998), response to positive end-expiratory pressure (PEEP) (Tugrul et al., 2003), response to PP (Lim et al., 2001), and radiographic imaging on chest CT (Goodman et al., 1999). The heterogeneity of ARDS complicates its diagnosis and treatment. Consequently, it is imperative to develop predictive models tailored to specific etiologies to enhance the accuracy and efficacy of clinical interventions.

Biomarkers are measurable indicators of normal or pathological processes or responses to therapeutic interventions, frequently used as auxiliary indices in clinical practice. Previous research has investigated the predictive and prognostic value of biomarkers in ARDS (Samanta et al., 2018; Leija-Martinez et al., 2020). ARDS can be induced by various pulmonary and extrapulmonary diseases, leading to distinct pathophysiological changes. Biomarkers may exhibit heterogeneity due to these differences in primary etiologies. For instance, biomarkers differ between pulmonary ARDS and extrapulmonary ARDS (Chen et al., 2013). Therefore, it is crucial to ensure that study populations are homogeneous when investigating ARDS biomarkers. Additionally, the pathogenesis of ARDS is extremely intricate, with various pathophysiological alterations frequently overlapping temporally and spatially. This overlap leads to considerable heterogeneity and variability in biomarkers (Cartin-Ceba et al., 2015), thereby constraining the relevance and value of research focusing on single biomarkers (Levitt and Rogers, 2016). Compared to single biomarkers, the combined use of multiple biomarkers is more advantageous for the early diagnosis, classification, and prognostic evaluation of ARDS (Cartin-Ceba et al., 2015; Bos et al., 2017; Negrin et al., 2017). However, studies on the combined use of biomarkers in ARDS are limited and have not yet reached clinical applicability (Ware et al., 2017; Ge et al., 2023). Additionally, some biomarkers are not routinely measured in clinical practice, and the feasibility of implementing multi-biomarker predictive methods in clinical settings is constrained. Therefore, targeting biomarkers most relevant to pathophysiology, combined with clinical diagnostic indicators and other clinical characteristic factors, may help predict and evaluate the prognosis of ARDS mediated by specific primary diseases and mechanisms.

This study aims to analyze patients with extrapulmonary ARDS, utilizing early clinical data that can be rapidly obtained to identify characteristic factors. These factors will be combined with protein biomarkers indicative of pulmonary endothelial injury and clinical diagnostic indicators reflecting pulmonary pathophysiological status. The objective of this study is to construct and validate a nomogram model to provide valuable references for the clinical diagnosis and treatment of ARDS.

## 2 Materials and methods

### 2.1 Participant recruitment

This investigation enrolled participants admitted to the ICUs at the Central Theater General Hospital of the People’s Liberation Army of China and Hefei BOE Hospital from June 2019 to June 2022. We included individuals meeting the following criteria: 1) diagnosed with moderate to severe ARDS according to the 2012 Berlin criteria, displaying a partial pressure of arterial oxygen to fractional inspired oxygen (PaO_2_/FiO_2_) ratio ≤ 200 mmHg under a standardized ventilation regime (Force et al., 2012); 2) ARDS originating from indirect lung injury causes, including but not limited to non-pulmonary sepsis, trauma not involving the lungs, adverse reactions to blood transfusions, pancreatitis, among others; 3) diagnosis of ARDS confirmed within the initial 24 h following ICU admission. The exclusion criteria encompassed: 1) age below 18 or above 85 years; 2) pregnancy; 3) history of pulmonary tuberculosis, lung cancer, severe chronic obstructive pulmonary disease (COPD), chronic interstitial lung disease, or prior lung surgery; 4) presence of massive pleural effusion or pneumothorax; 5) cardiogenic pulmonary edema or hypoxemia primarily due to cardiac conditions; 6) inadequate acoustic window for ultrasound imaging; 7) inability to ascertain clinical outcomes within the study’s observation period.

This study was conducted in strict accordance with ethical principles and received approval from the review boards of the two participating hospital institutions (Approval No: 201910216; 20190010). Prior to enrollment, written informed consent was obtained from the legal guardians of the patients. All patient information was anonymized before analysis to ensure confidentiality.

### 2.2 Definitions and diagnostic criteria

ARDS was identified following the Berlin Definition criteria, which necessitate: an acute inception, evident bilateral infiltrates on chest radiographs not fully explained by effusions, lobar/lung collapse, or nodules; a non-cardiogenic origin as indicated by the absence of left atrial hypertension; and a PaO_2_/FiO_2_ ratio less than 300 mmHg (Force et al., 2012). Distinctively, extrapulmonary ARDS results from systemic factors that precipitate vascular endothelial damage, enhance pulmonary vascular permeability, lead to interstitial and alveolar exudation, and culminate in alveolar collapse, edema, and respiratory failure (Bernard et al., 1994).

### 2.3 Data collection on clinical characteristics and initial management

Upon enrollment, we systematically collected comprehensive baseline clinical data for each participant. This encompassed demographic and physiological characteristics, the etiological factors contributing to ARDS, and any pre-existing health conditions. Within the first 24 h post-enrollment, we meticulously recorded vital signs, laboratory findings, assessed the severity of the disease using the Acute Physiology and Chronic Health Evaluation (APACHE) II scoring system, and determined the degree of oxygenation impairment via the PaO_2_/FiO_2_ ratio. These initial assessments provided insight into the patients’ baseline health status and the immediate therapeutic interventions initiated upon study entry.

Demographic data included age, sex, body mass index (BMI), and smoking history. The causative factors of ARDS were categorized into distinct groups: non-pulmonary sepsis, non-pulmonary trauma, complications arising from multiple blood transfusions, pancreatitis, and other causes. We also compiled an extensive list of pre-existing conditions, such as hypertension, coronary artery disease, diabetes mellitus, and cerebrovascular diseases.

Vital signs included mean arterial pressure, heart rate, respiratory rate, and body temperature. The laboratory parameters assessed encompassed hemoglobin concentration, white blood cell count, platelet count, serum creatinine, total bilirubin, albumin levels, troponin T, NT-proBNP, D-dimer, lactate, procalcitonin, C-reactive protein, and electrolytes (sodium and potassium). The APACHE II score was calculated to assess the initial severity of the illness (Knaus et al., 1985), while the PaO_2_/FiO_2_ ratio (Force et al., 2012) provided an index of the baseline level of pulmonary oxygenation dysfunction.

Therapeutic interventions initiated within the first 24 h of patient enrollment were meticulously recorded. These included the application of mechanical ventilation, the adoption of PP, the administration of vasoactive drugs, sedation, neuromuscular blocking agents, systemic corticosteroids, antibiotics, antifungal treatments, continuous renal replacement therapy, and venovenous ECMO (VV ECMO). These management strategies were individualized based on each patient’s clinical status and underlying conditions, in alignment with current best practice guidelines and clinical protocols.

### 2.4 Management of respiratory failure and supportive care in ARDS

All study participants received care in the ICU adhering to globally recognized ARDS management guidelines and consensus documents. Initial respiratory support was provided using non-invasive or invasive mechanical ventilation (NIV or IMV, respectively) upon patient enrollment. Transition from NIV to IMV was considered under specific conditions such as a high Simplified Acute Physiological Score (>34), PaO_2_/FiO_2_ ratio ≤ 175 mmHg (indicating severe hypoxemia) (Antonelli et al., 2007), lack of hypoxemia improvement after 1 h of NIV, or excessive spontaneous breathing leading to large tidal volumes (>9 mL/kg) (Frat et al., 2018).

For patients on IMV, a lung-protective ventilation strategy was employed aiming to optimize oxygenation and minimize ventilator-induced lung injury. Specific targets included maintaining peripheral capillary oxygen saturation between 88% and 95%, PaO_2_ levels of 55–80 mmHg, partial pressure of arterial carbon dioxide (PaCO_2_) ≤ 50 mmHg or pH ≥ 7.25, plateau pressure (Pplat) ≤ 30 cmH_2_O, and driving pressure (∆P) ≤ 15 cmH_2_O (Khemani et al., 2018). Tidal volume was initially set at 6 mL/kg of predicted body weight and adjusted based on Pplat and ∆P assessments. PEEP levels were set according to FiO_2_ requirements and subsequently fine-tuned based on Pplat, ∆P, and PaCO_2_ levels. Respiratory rate adjustments were made to manage PaCO_2_ and pH levels effectively. PP was implemented for durations exceeding 12 h per day in cases of persistent acidosis (pH < 7.25), PaCO_2_ > 50 mmHg, or when the PaO_2_/FiO_2_ ratio was ≤150 mmHg despite a PEEP ≥ 5 cmH_2_O, unless contraindicated (Fan et al., 2017; Fichtner et al., 2018). Neuromuscular blockade was utilized in scenarios of unresolving hypoxemia, during PP, or to prevent ventilator-induced injuries, in line with recent rapid practice guidelines (Alhazzani et al., 2020).

VV ECMO was considered for patients with severe respiratory failure unresponsive to mechanical ventilation if specific criteria were met, including a treatment duration under 7 days with FiO_2_ ≥ 0.80, a PaO_2_/FiO_2_ ratio < 50 mmHg for over 3 h or <80 mmHg for over 6 h, pH < 7.25 with PaCO_2_ ≥ 60 mmHg for more than 6 h, respiratory rates ≥ 35 min, or Pplat ≤ 32 cmH_2_O (Tonna et al., 2021).

Fluid management and vasoactive drug administration were tailored by the attending physician based on hemodynamic status, ARDS severity, and cardiac and renal function. In cases of concurrent sepsis, fluid and vasopressor strategies followed sepsis-specific guidelines (Singer et al., 2016). Corticosteroid use adhered to guidelines addressing critical illness-associated corticosteroid insufficiency and international sepsis guidelines (Singer et al., 2016; Pastores et al., 2018). Additionally, nutritional support and venous thromboembolism prophylaxis were provided in accordance with critical care guidelines and consensus statements (Di Nisio et al., 2016; Burgos et al., 2018).

### 2.5 Lung ultrasound score (LUS) assessment protocol

LUS examinations were conducted within the initial 24 h post-enrollment. The pulmonary regions were systematically partitioned into twelve segments via anatomical delineations, which included the parasternal, anterior axillary, posterior axillary, posterior median lines, and the interscapular line. Utilizing a phased array convex transducer (frequency range: 3.5–10 MHz, manufactured by GE Healthcare, United States), scanning was meticulously performed across these predefined segments. Pulmonary ultrasound findings were quantified based on a four-point scale reflecting the extent of ventilatory compromise: 1) Score 0 indicating normal aeration evidenced by lung sliding and the presence of A-lines or fewer than three discrete B-lines; 2) Score 1 for moderate aeration loss, characterized by the appearance of three or more scattered B-lines; 3) Score 2 denoting severe ventilatory impairment with confluent B-lines; 4) Score 3 corresponding to pulmonary consolidation, identifiable by tissue-like echotexture and bronchogram signs. A composite score ranging from 0 to 36 was then computed, providing an aggregate measure of pulmonary aeration deficit (Mongodi et al., 2017).

### 2.6 Quantification of serum soluble thrombomodulin (sTM) levels

Serum samples for sTM quantification were obtained within the first 24 h following patient enrollment. Concentrations of sTM were determined employing a dual-antibody sandwich enzyme-linked immunosorbent assay, specifically the kit supplied by Abcam (catalogue number ab46508, Shanghai, China). All procedures were rigorously conducted in adherence to the manufacturer-provided protocol.

### 2.7 Primary outcome determination and patient stratification

The principal outcome was assessed as all-cause mortality within a 28-day interval post-admission. Patients were subsequently categorized into two distinct cohorts predicated on their 28-day survival outlook: those who survived (survival group) and those who did not (non-survival group).

### 2.8 Statistical analysis

Data analyses were executed using standardized statistical methodologies. Continuous variables underwent normality testing through the Shapiro-Wilk test, alongside Bartlett’s test for assessing variance homogeneity. Variables adhering to normal distribution were articulated as mean ± standard deviation, with inter-group discrepancies evaluated via the independent samples t-test. Conversely, variables deviating from normal distribution were presented as median (interquartile range, IQR), with the Mann-Whitney U test facilitating inter-group comparisons. Categorical variables were represented as percentages (%) and analyzed using the chi-square test. Univariate Cox regression analysis was employed for variables deemed clinically pertinent and demonstrating significant variances between survival outcomes. Lasso regression was utilized for risk factor identification, with the Schoenfeld residual test confirming the proportional hazard (PH) assumption post Lasso-Cox regression. A prognostic nomogram was formulated based on Lasso-Cox regression outcomes, its predictive accuracy gauged through the concordance index (C-index), calibration plots, internal validation, decision curve analysis (DCA), interventions avoided analysis (IAA), and receiver operating characteristic (ROC) analysis. The area under the ROC curve (AUC) was used to determine the predictive accuracy of each factor, and the DeLong test was employed to compare the AUCs. Patient survival probabilities were delineated via the Kaplan-Meier method. Data processing was accomplished using SPSS version 22.0 and STATA version 17.0, with statistical significance set at a *p*-value < 0.05 (two-tailed).

## 3 Results

### 3.1 Characteristics of the study cohort

**FIGURE 1:**
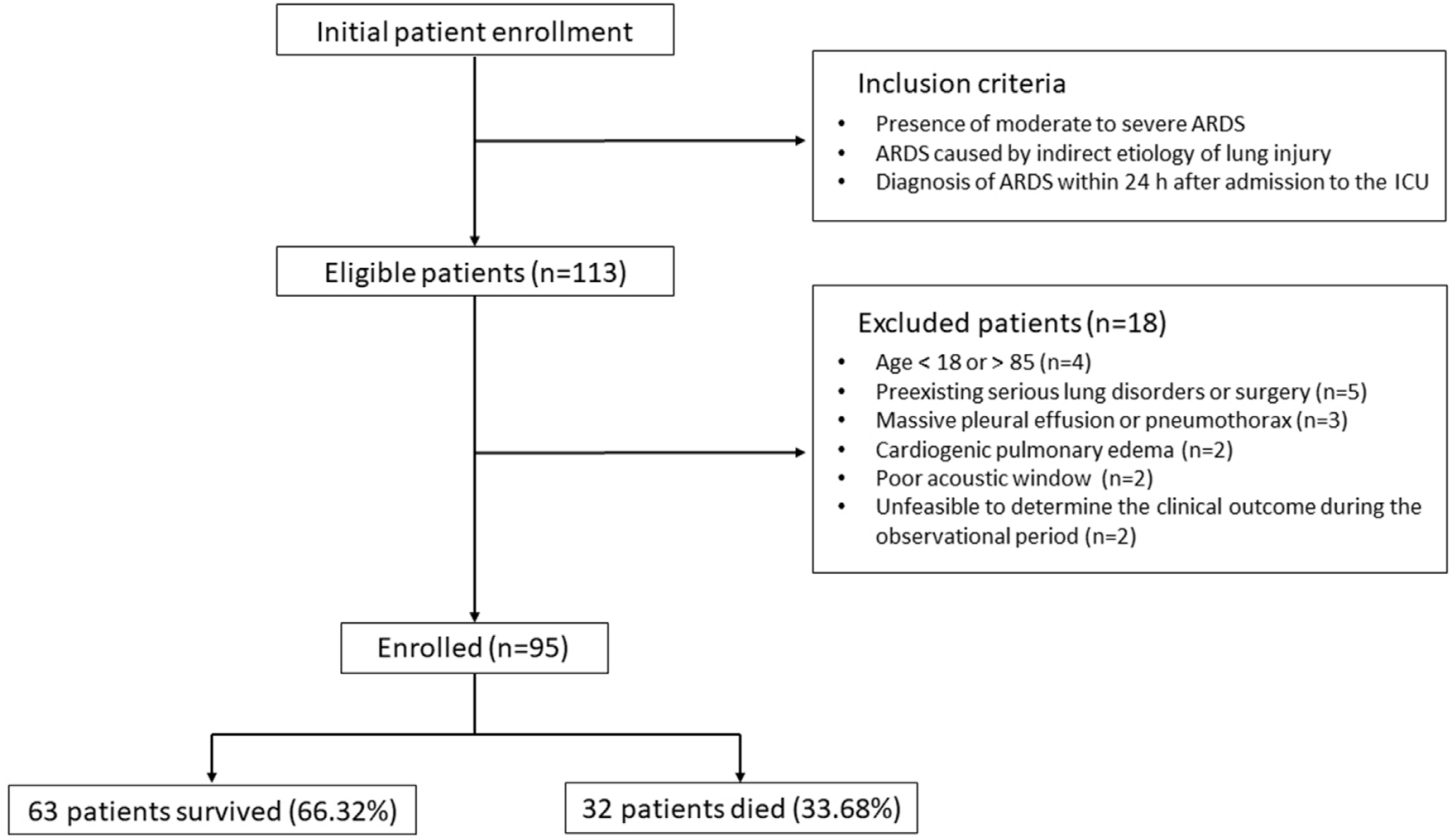
Flowchart for patient enrollment and exclusion.

During the recruitment phase, 113 individuals were identified as eligible according to the inclusion criteria, as depicted in Figure 1. However, 18 participants were subsequently excluded due to reasons including age constraints (below 18 or above 85 years), the presence of severe pre-existing pulmonary conditions or recent thoracic surgeries, significant pleural effusion or pneumothorax, cardiogenic pulmonary edema, inadequate echogenicity for ultrasound examination, and the inability to ascertain clinical outcomes within the study’s observational timeframe. Consequently, the study proceeded with 95 participants, comprising 32 individuals in the non-survival cohort (33.68%) and 63 in the survival cohort (66.32%).

### 3.2 Comparative analysis of clinical and laboratory parameters

#### 3.2.1 Demographic and clinical characteristics

The baseline demographic and clinical characteristics, including the etiology of ARDS and pre-existing health conditions, are summarized in Table 1. Comparative analysis revealed no statistically significant disparities between survivors and non-survivors concerning age, gender distribution, BMI, and smoking history (*p* > 0.05 for all comparisons). Similarly, the underlying causes of ARDS, spanning non-pulmonary sepsis, trauma, multiple transfusions, pancreatitis, among others, showed no significant variance between the two groups (*p* > 0.05 for all etiologies). Furthermore, the prevalence of pre-existing conditions such as hypertension, coronary artery disease, diabetes, and cerebrovascular diseases was comparable between survivors and non-survivors (*p* > 0.05 for all conditions).

**TABLE 1:** General characteristics, ARDS etiology, and preexisting medical conditions of patients with extrapulmonary ARDS between survivors and non-survivors.

Variables	Survivors (n = 63)	Non-survivors (n = 32)	*p*-value
General characteristics (mean ± SD) or (n, %)			
Age (years)	49.94 ± 11.23	51.06 ± 12.84	0.661
Male	40 (63.49)	24 (75.00)	0.258
BMI (kg/m^2^)	25.39 ± 2.94	25.86 ± 2.99	0.461
Smoking	19 (30.26)	8 (25.00)	0.598
ARDS etiology (n, %)			
Non-pulmonary sepsis	33 (52.38)	17 (53.13)	0.945
Non-pulmonary trauma	12 (19.05)	8 (25.00)	0.501
Multiple transfusion	7 (11.11)	3 (9.38)	0.794
Pancreatitis	5 (7.94)	2 (6.25)	0.766
Other	6 (9.52)	2 (6.25)	0.587
Preexisting medical conditions (n, %)			
Hypertension	15 (23.81)	6 (18.75)	0.574
Coronary atherosclerotic heart disease	9 (14.29)	6 (18.75)	0.573
Diabetes	12 (19.06)	5 (15.63)	0.681
Cerebrovascular disease	4 (6.35)	3 (9.38)	0.594

n, number; SD, standard deviation; BMI, body mass index; Other, non-pulmonary high-risk surgery, post-cardiopulmonary bypass, neurological disease, cardiac arrest, burn.

#### 3.2.2 Vital signs, laboratory findings and clinical scores

Table 2 elucidates the vital signs, laboratory findings, APACHE II scores, and PaO_2_/FiO_2_ ratios assessed within the initial 24 h post-enrollment. Noteworthy disparities were observed in platelet count (184.30 vs. 141.15, *p* = 0.017), serum creatinine levels (153.10 vs. 182.76, *p* = 0.046), and lactate levels (4.20 vs. 5.35, *p* = 0.001) between survivors and non-survivors, indicating a significantly more compromised physiological status in the latter group. Additionally, the APACHE II scores were significantly lower (21.52 ± 2.68 vs. 23.28 ± 2.63, *p* = 0.003), and PaO_2_/FiO_2_ ratios were notably higher (119.14 ± 14.02 vs. 109.47 ± 11.04, *p* = 0.001) in the survival cohort, suggesting a less severe clinical presentation and better oxygenation status at baseline.

**TABLE 2:** Vital signs, laboratory findings, APACHE II score, PaO_2_/FiO_2_ ratio, and treatments of patients with extrapulmonary ARDS between survivors and non-survivors.

Variables	Survivors (n = 63)	Non-survivors (n = 32)	*p*-value
Vital signs			
Mean arterial pressure (mmHg)	82 ± 15	79 ± 13	0.345
Heart arte (bpm)	84 ± 11	86 ± 11	0.325
Respiratory rate (bpm)	25 ± 7	26 ± 8	0.489
Temperature (°C)	38.0 ± 1.0	38.3 ± 1.0	0.183
Laboratory findings within 24 h of enrollment (mean ± SD) or (median, IQR)			
Hemoglobin level (g/L)	107.14 ± 25.10	100.97 ± 23.11	0.248
Leucocytes count (109/L)	13.78 ± 3.17	15.05 ± 3.25	0.070
Platelet count (10^9^/L)	184.30 (134.10,217.30)	141.15 (103.03,184.60)	0.017*
Creatinine (μmol/L)	153.10 (109.00, 219.62)	182.76 (139.01,255.50)	0.046*
Total bilirubin (μmol/L)	24.60 (17.60, 33.20)	30.80 (19.50, 38.49)	0.104
Albumin (g/L)	27.71 ± 6.08	25.26 ± 5.78	0.062
Troponin T (μg/L)	5.26 (3.14, 7.83)	6.68 (3.80, 9.18)	0.145
N-terminal pro-B-type natriuretic peptide (pg/mL)	822.00 (519.00, 3146.00)	912.00 (449.75, 4362.50)	0.671
D-dimer (mg/L)	4.91 ± 1.77	5.70 ± 2.05	0.055
Lactate (mmol/L)	4.20 (2.80,5.30)	5.35 (4.13,7.30)	0.001*
Procalcitonin (ng/mL)	3.88 (2.42, 6.70)	6.05 (2.94,13.19)	0.085
C-reactive protein (mg/L)	105.30 (73.40, 142.80)	123.05 (79.00, 174.13)	0.176
Sodium (mmol/L)	142.30 (136.00, 149.30)	140.40 (135.60, 147.70)	0.262
Potassium (mmol/L)	4.06 (3.34, 4.75)	4.12 (3.53, 4.73)	0.711
Severity of illness within 24 h of enrollment (mean ± SD)			
APACHE II score	21.52 ± 2.68	23.28 ± 2.63	0.003*
Lung injury within 24 h of enrollment (mean ± SD)			
PaO_2_/FiO_2_ ratio	119.14 ± 14.02	109.47 ± 11.04	0.001*
Treatments within 24 h of enrollment (n, %)			
Methods of respiratory support			
NIV	4 (6.35)	2 (6.25)	0.985
IMV	59 (93.65)	30 (93.75)	0.985
PP	16 (25.40)	11 (34.38)	0.359
Vasoactive agents	41 (65.08)	24 (75.00)	0.326
Sedation	51 (80.95)	28 (87.50)	0.420
Neuromuscular blockade	6 (9.52)	5 (15.63)	0.380
Systemic corticosteroids	1 (1.59)	2 (6.25)	0.219
Antibiotics	34 (53.97)	20 (62.50)	0.427
Antifungal	1 (1.59)	1 (3.12)	0.622
Continuous blood purification	3 (4.76)	3 (9.38)	0.382
VV ECMO	0	0	/

n, number; bpm, beats per minute; SD, standard deviation; IQR, interquartile range; APACHE II score, Acute Physiology and Chronic Health Evaluation II score; PaO_2_/FiO_2_ ratio, partial pressure of arterial oxygen to fractional inspired oxygen ratio; NIV, noninvasive positive pressure mechanical ventilation; IMV, invasive positive pressure mechanical ventilation; PP, prone positioning; VV ECMO, venovenous extracorporeal membrane oxygenation. The symbol * indicates *p* < 0.05.

#### 3.2.3 Treatment interventions

An in-depth comparison of therapeutic interventions administered within the first 24 h of enrollment is presented in Table 2. There was no statistical significance in the application of mechanical ventilation, positive pressure support, vasoactive agents, sedation, neuromuscular blockade, systemic corticosteroids, antibiotic and antifungal treatments, continuous blood purification, and VV ECMO between the two groups (*p* > 0.05 for all treatment modalities).

### 3.3 Comparison of sTM levels and LUS between survivors and non-survivors

The baseline levels of sTM and LUS were compared between patients who survived and those who did not survive in order to investigate potential differences related to patient outcomes. Table 3 illustrates that survivors exhibited significantly lower serum sTM levels and LUS compared to non-survivors (all *p* < 0.05).

**TABLE 3:** sTM levels and LUS of patients with extra-pulmonary ARDS between survivors and non-survivors.

Variables (mean ± SD)	Survivors (n = 63)	Non-survivors (n = 32)	*p*-value
sTM (ng/mL)	93.71 ± 22.04	118.72 ± 30.97	<0.001*
LUS	13.30 ± 2.91	16.03 ± 3.03	<0.001*

SD, standard deviation; n, number; sTM, serum soluble thrombomodulin; LUS, lung ultrasound score. The symbol * indicates *p* < 0.05.

### 3.4 Identification of independent predictors for 28-day mortality in patients

Univariate Cox regression analysis was conducted to identify potential predictors for 28-day mortality. The analysis revealed that sTM level [odds ratio (OR) = 1.030, 95% confidence interval (CI) 1.018–1.042, *p* < 0.001], LUS (OR = 1.255, 95% CI 1.122–1.404, *p* < 0.001), APACHE II score (OR = 1.232, 95% CI 1.073–1.415, *p* = 0.003), PaO_2_/FiO_2_ ratio (OR = 0.959, 95% CI 0.934–0.985, *p* = 0.002), platelet count (OR = 0.994, 95% CI 0.988–0.999, *p* = 0.028), creatinine (OR = 1.005, 95% CI 1.001–1.009, *p* = 0.026), and lactate (OR = 1.212, 95% CI 1.073–1.369, *p* = 0.002) were significantly associated with 28-day mortality (Table 4).

**TABLE 4:** Univariate Cox regression analysis of risk factors for 28-day mortality in patients with extra-pulmonary ARDS.

Variables	β	S.E.	Waldχ^2^	OR	95% CI	*p*-value
sTM (ng/mL)	0.029	0.006	25.316	1.030	1.018–1.042	<0.001*
LUS	0.227	0.057	15.760	1.255	1.122–1.404	<0.001*
Age (years)	0.006	0.016	0.123	1.006	0.975–1.038	0.726
APACHE II score	0.209	0.071	8.788	1.232	1.073–1.415	0.003*
PaO_2_/FiO_2_ ratio	−0.042	0.014	9.523	0.959	0.934–0.985	0.002*
Non-pulmonary sepsis	0.123	0.356	0.119	1.131	0.562–2.274	0.730
Platelet count (10^9^/L)	−0.006	0.003	4.809	0.994	0.988–0.999	0.028*
Creatinine (μmol/L)	0.005	0.002	4.929	1.005	1.001–1.009	0.026*
Lactate (mmol/L)	0.192	0.065	9.596	1.212	1.073–1.369	0.002*

S.E., standard error; OR, odds ratio; CI, confidence interval; sTM, serum soluble thrombomodulin; LUS, lung ultrasound score; APACHE II score, Acute Physiology and Chronic Health Evaluation II score; PaO_2_/FiO_2_ ratio, partial pressure of arterial oxygen to fractional inspired oxygen ratio. The symbol * indicates *p* < 0.05.

**FIGURE 2:**
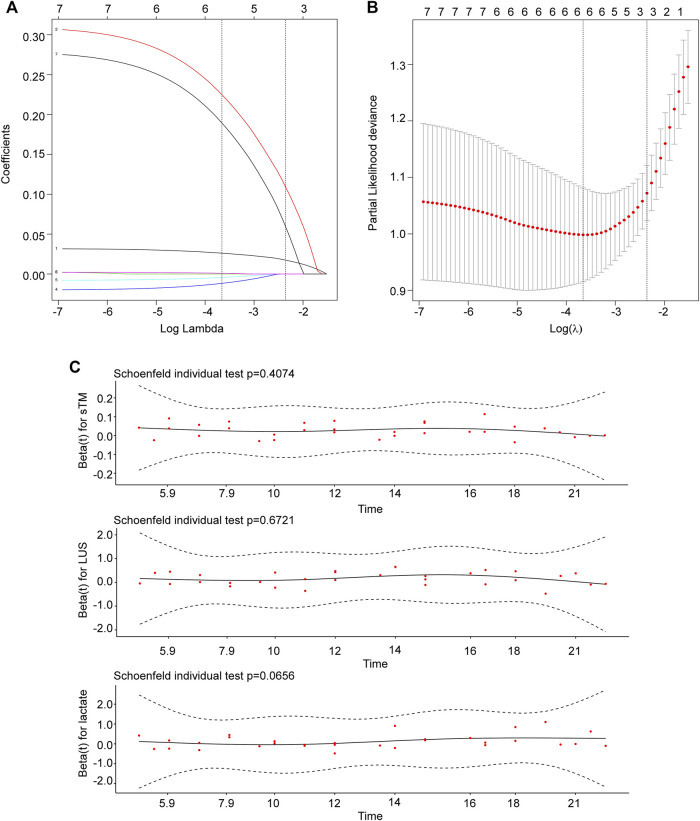
Identification and screening of prognostic variables. **(A)** The parameter path was estimated over a range of values for λ. **(B)** The selection process of the optimal value of the parameter λ in the Lasso regression model using cross-validation. The number of variables selected was three. **(C)** sTM, LUS, and lactate met the PH assumption. sTM, serum soluble thrombomodulin; LUS, lung ultrasound score.

Subsequently, Lasso regression analysis was employed to further refine the predictive variables. The parameter path was estimated over a range of values for λ, as depicted in Figure 2A. Through iterative analysis and 10-fold crossvalidation, the optimal value of λ was determined to be 0.095, resulting in a model with exceptional performance and minimal variables (Figure 2B). The final screened variables included sTM, LUS, and lactate.

To ensure the validity of the model, the baseline levels of sTM, LUS, and lactate were subjected to the proportional risk hypothesis test based on Schoenfeld residuals (Figure 2C). The results showed that all variables met the PH assumptions (sTM, *p* = 0.407; LUS, *p* = 0.672; lactate, *p* = 0.066). Therefore, all three variables were utilized in the predictive modeling for 28-day mortality.

### 3.5 Development and validation of the integrated predictive nomogram

**FIGURE 3:**
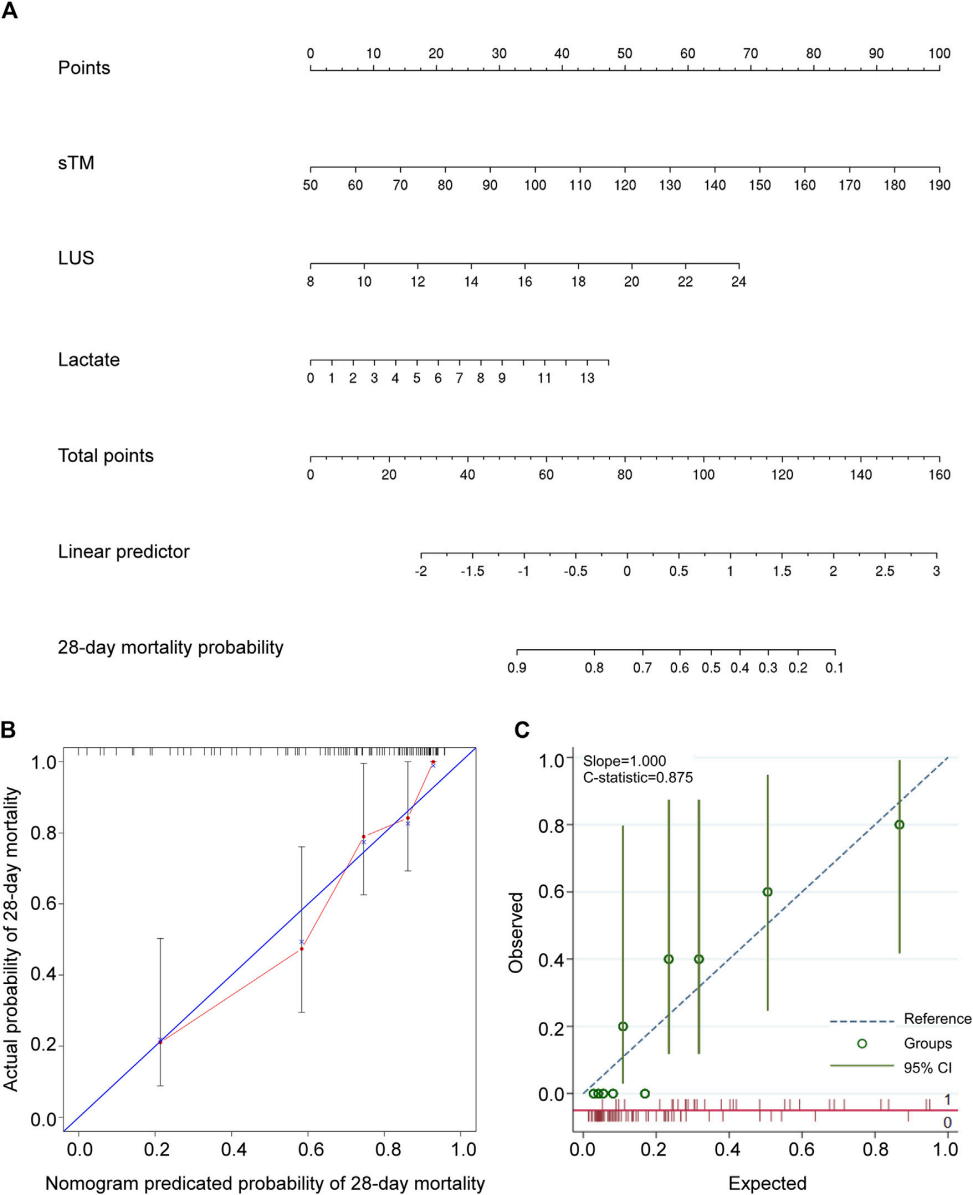
Development and validation of a predictive nomogram for forecasting 28-day overall survival. **(A)** The nomogram for predicting the 28-day survival probability of patients with extrapulmonary ARDS. The nomogram comprised three variables, namely sTM, LUS, and lactate. **(B)** The calibration curve of the nomogram. **(C)** The internal validation of the nomogram. sTM, serum soluble thrombomodulin; LUS, lung ultrasound score; CI, confidence interval.

The constructed nomogram visually represents the impact of sTM, LUS, and lactate on patient survival outcomes. The “total points” projection provides an estimation of the probability of 28-day mortality based on the combination of these variables (Figure 3A), facilitating informed clinical decision-making. The median C-index for the nomogram was 0.807 (0.740–0.873), indicating excellent predictive accuracy. Calibration curve analysis demonstrated strong agreement between predicted and observed outcomes (Figure 3B), reinforcing the reliability of the nomogram. Internal validation of the nomogram yielded a C-index of 0.875, confirming its robustness (Figure 3C).

**FIGURE 4:**
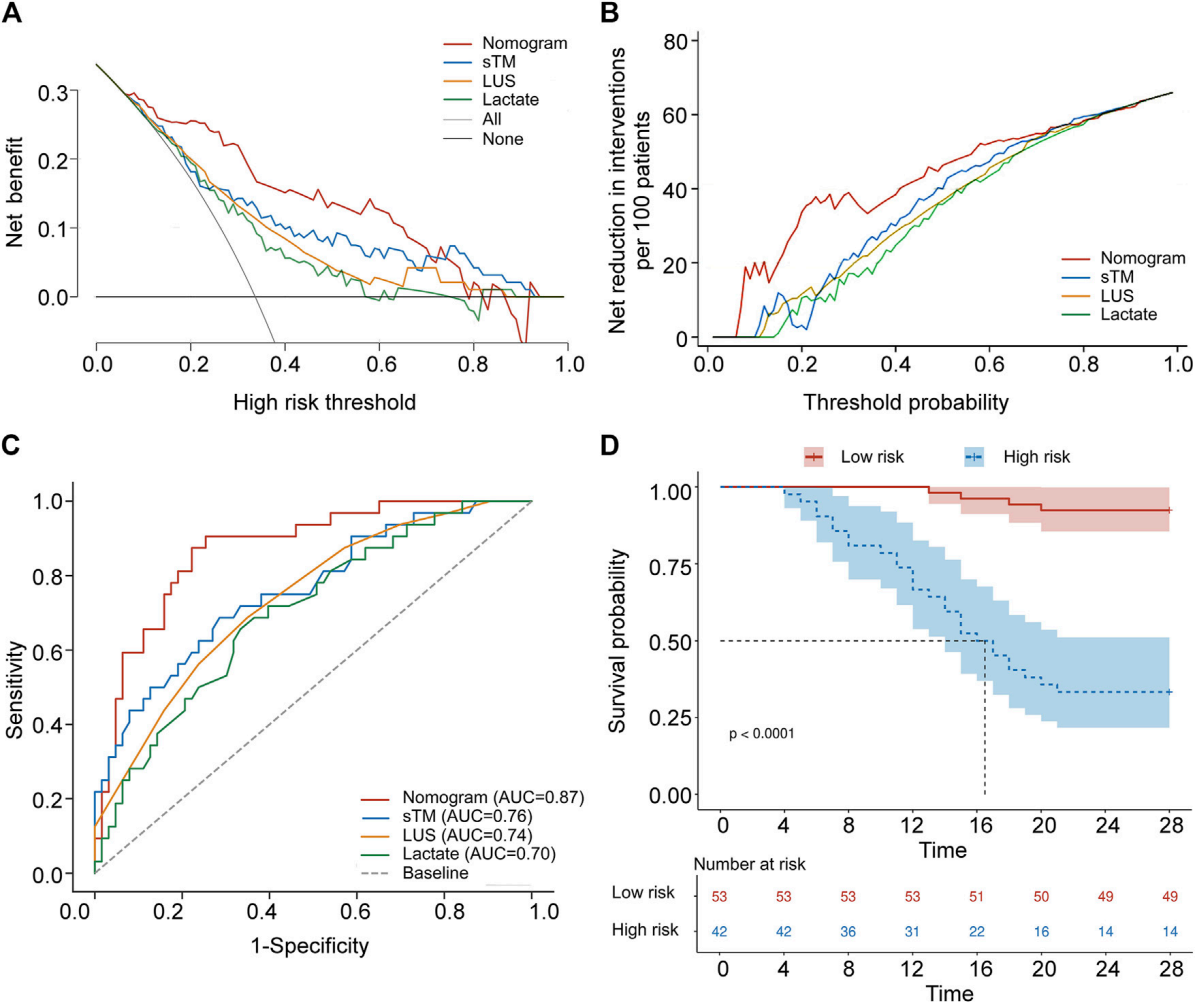
Validation of a predictive nomogram for forecasting 28-day overall survival. **(A)** Decision curve analysis of sTM, LUS, lactate, and the nomogram. **(B)** Analysis of avoided interventions for sTM, LUS, lactate, and the nomogram. **(C)** ROC curves of sTM, LUS, lactate, and the nomogram for predicting 28-day mortality. **(D)** Kaplan-Meier 28-day survival curve of patients with varying cut-off values of the nomogram. sTM, serum soluble thrombomodulin; LUS, lung ultrasound score; AUC, area under curve.

To evaluate the clinical utility of the predictive model, DCA and IAA were performed. Figure 4A illustrates the clinical effectiveness of the nomogram predictive model as assessed by DCA, demonstrating a higher net benefit for clinical decision-making within a threshold probability range of 0–0.71. Figure 4B shows that the IAA analysis indicates a net reduction in unnecessary interventions within a threshold probability range of 0–0.73. Table 5 presents the ROC analysis of the model’s predictive performance. The nomogram demonstrated significantly superior predictive ability for 28-day mortality compared to individual factors such as sTM, LUS, and lactate, with an AUC of 0.873 (95% CI 0.789–0.932). The DeLong test confirmed that the AUC differences between the nomogram and each individual factor were statistically significant (all *p* < 0.05). Additionally, the nomogram exhibited higher sensitivity (87.50%) and specificity (77.78%) in predicting 28-day mortality compared to these individual factors. Figure 4C visually illustrates the AUC values obtained from the ROC analysis. Furthermore, the Kaplan-Meier survival analysis, depicted in Table 6, indicated a significantly lower overall survival rate in the high-risk group compared to the low-risk group (66.67% vs. 7.55%, *p* < 0.001). The mean survival time was also substantially shorter for the high-risk group (17.69 days vs. 27.13 days, 95% CI 15.19–20.19 days vs. 26.29–27.97 days, *p* < 0.001). Figure 4D illustrates the Kaplan-Meier survival curves for the high-risk and low-risk groups.

**TABLE 5:** Youden index analyses of sTM, LUS, lactate, and the nomogram for predicting 28-day mortality.

	AUC	95% CI	S.E.	Z statistics	*p*-value	Cut-off value	Sensitivity (%)	Specificity (%)
sTM	0.760	0.662–0.842	0.053	4.921	<0.001*	>104.52 (ng/mL)	68.75	71.43
LUS	0.737	0.637–0.822	0.053	4.492	<0.001*	>13.50	68.75	65.08
Lactate	0.702	0.600–0.792	0.056	3.638	<0.001*	>4.63 (mmol/L)	65.62	66.67
Nomogram	0.873	0.789–0.932	0.037	10.019	<0.001*	>0.31	87.50	77.78

AUC, area under curve; CI, confidence interval; S.E., standard error; sTM, serum soluble thrombomodulin; LUS, lung ultrasound score. The symbol * indicates *p* < 0.05.

**TABLE 6:** Comparison of 28-day mortality and survival status in patients with varying cut-off values of the nomogram.

	Patients (n = 95)	Death (n = 32)	28-day mortality (%)	Mean survival day (days)	95% CI (days)
Nomogram ≤ 0.31	53	4	7.55	27.13	26.29–27.97
Nomogram > 0.31	42	28	66.67	17.69	15.19–20.19

n, number; CI, confidence interval.

### 3.6 Evaluation of the predictive accuracy of the integrated predictive nomogram

In clinical practice, the APACHE II score is a widely accepted prognostic tool for assessing the severity of critical illness, while the PaO_2_/FiO_2_ ratio is a reliable indicator for evaluating the severity of ARDS. This study conducted a comparative analysis to assess the predictive accuracy of the combined nomogram versus traditional models, particularly the APACHE II score and PaO_2_/FiO_2_ ratio.

**FIGURE 5:**
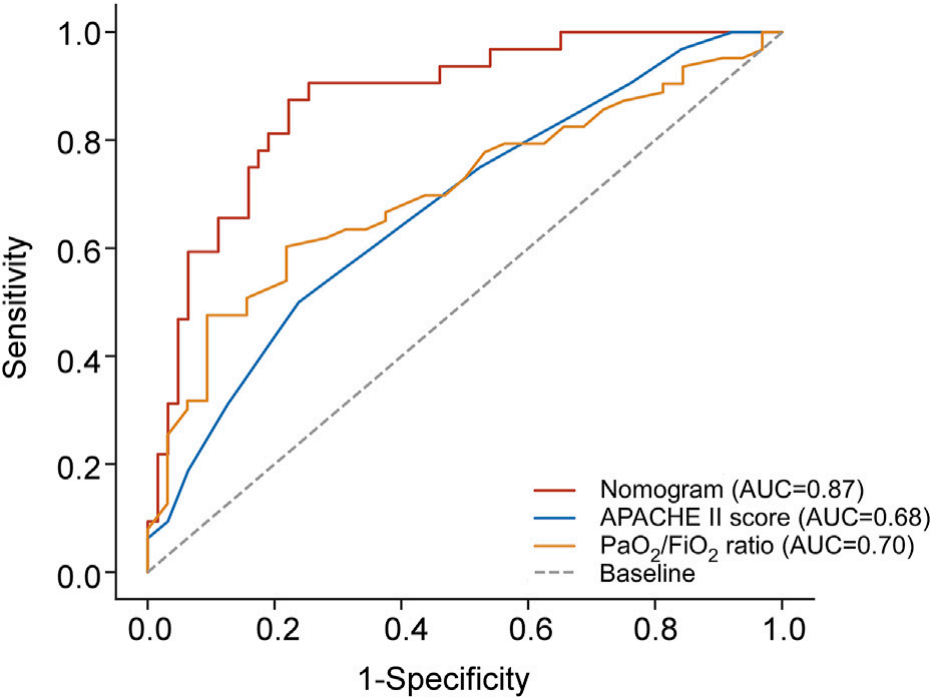
Comparison of AUC between the combined predictive nomogram and the traditional predictive models, including APACHE II score and PaO_2_/FiO_2_ ratio. APACHE II score, Acute Physiology and Chronic Health Evaluation II score; PaO_2_/FiO_2_ ratio, partial pressure of arterial oxygen to fractional inspired oxygen ratio; AUC, area under curve.

ROC analysis was employed to evaluate and compare the predictive accuracy of these models. As shown in Table 7, the nomogram achieved a higher AUC than both the APACHE II score (AUC 0.676, 95% CI 0.572–0.768) and the PaO_2_/FiO_2_ ratio (AUC 0.704, 95% CI 0.602–0.794). The DeLong test confirmed that the differences in AUC between the nomogram and each individual factor were statistically significant (all *p* < 0.05). Additionally, the nomogram showed superior sensitivity and specificity for predicting 28-day mortality compared to the APACHE II score (sensitivity 50.00%, specificity 76.19%) and the PaO_2_/FiO_2_ ratio (sensitivity 78.12%, specificity 60.32%). Figure 5 provides a visual representation of the AUC values for these models.

**TABLE 7:** Youden index analyses of APACHE II score, PaO_2_/FiO_2_ ratio, and nomogram for predicting 28-day mortality in patients with extra-pulmonary ARDS.

Variables	AUC	95% CI	S.E.	Z statistics	*p*-value	Cut-off value	Sensitivity (%)	Specificity (%)
APACHE II score	0.676	0.572–0.768	0.058	3.032	0.002*	>23	50.00	76.19
PaO_2_/FiO_2_ ratio	0.704	0.602–0.794	0.054	3.779	0.002*	≤116	78.12	60.32
Nomogram	0.873	0.789–0.932	0.037	10.019	<0.001*	>0.31	87.50	77.78

AUC, area under curve; CI, confidence interval; S.E., standard error; APACHE II score, acute physiology and chronic health evaluation II score; PaO_2_/FiO_2_ ratio, partial pressure of arterial oxygen to fractional inspired oxygen ratio. The symbol * indicates *p* < 0.05.

### 3.7 Subgroup analysis evaluating the role of sTM, LUS, and lactate in patients undergoing PP

The efficacy of PP in enhancing the clinical outcomes of patients afflicted with ARDS has been well-documented in the literature (Guerin et al., 2013). Nonetheless, the introduction of medical interventions during the observation period can potentially introduce confounding factors, thereby affecting the predictive accuracy of our combined nomogram. To mitigate this issue and elucidate the specific impact of PP on clinical outcomes, we conducted a subgroup analysis, categorizing patients based on their exposure to PP during the study period. Patients were thus classified into two groups: those who did not undergo PP treatment (non-PP group) and those who received PP treatment (PP group).

At the commencement of the study, comparative analysis of baseline characteristics, specifically sTM, LUS, and lactate levels, between the PP and non-PP groups revealed no statistically significant differences, as delineated in Table 8. Subsequent stratification based on PP exposure facilitated a more nuanced analysis of the relationship between these biomarkers and patient outcomes. Table 9 delineates the differential baseline levels of sTM, LUS, and lactate between patients who survived and those who did not, following stratification by PP status.

**TABLE 8:** sTM, LUS, and lactate of patients with extra-pulmonary ARDS between non-PP and PP patients.

Variables (mean ± SD)	Non-PP patients (n = 68)	PP patients (n = 27)	*p*-value
sTM (ng/mL)	100.24 ± 26.67	106.93 ± 30.76	0.294
LUS	14.13 ± 2.94	14.44 ± 3.86	0.671
Lactate (mmol/L)	4.77 ± 2.32	5.04 ± 2.46	0.614

SD, standard deviation; PP, prone positioning; n, number; sTM, serum soluble thrombomodulin; LUS, lung ultrasound score.

**TABLE 9:** Comparison of sTM, LUS and lactate in subgroups.

Subgroups	Variables		Mean ± SD	*p*-value
Non-PP patients (n = 68)	sTM (ng/mL)	Survivors (n = 47)	92.96 ± 21.86	<0.001*
		Non-survivors (n = 21)	116.52 ± 29.67	
	LUS	Survivors (n = 47)	13.64 ± 2.94	0.037*
		Non-survivors (n = 21)	15.24 ± 2.68	
	Lactate	Survivors (n = 47)	4.23 ± 2.08	0.003*
		Non-survivors (n = 21)	5.97 ± 2.43	
PP patients (n = 27)	sTM (ng/mL)	Survivors (n = 16)	95.94 ± 23.14	0.022*
		Non-survivors (n = 11)	122.91 ± 34.40	
	LUS	Survivors (n = 16)	12.31 ± 2.65	<0.001*
		Non-survivors (n = 11)	17.55 ± 3.21	
	Lactate (mmol/L)	Survivors (n = 16)	4.33 ± 1.76	0.070
		Non-survivors (n = 11)	6.07 ± 3.02	

SD, standard deviation; n, number; sTM, serum soluble thrombomodulin; LUS, lung ultrasound score. The symbol * indicates *p* < 0.05.

Moreover, Table 10 articulates the findings derived from both univariate and multivariate Cox regression analyses, examining the association between sTM, LUS, lactate levels, and 28-day mortality subsequent to stratification by PP. These analyses were meticulously adjusted for potential confounders, including the APACHE II score, PaO_2_/FiO_2_ ratio, platelet count, and creatinine levels. Intriguingly, our analysis revealed that, upon adjusting for these confounding factors, the associations of sTM, LUS, and lactate levels with 28-day mortality were no longer statistically significant among patients stratified by PP.

**TABLE 10:** Univariate and multivariate Cox regression analysis of sTM, LUS and lactate for predicting 28-day mortality in subgroups.

Subgroups	Variable		β	S.E.	Waldχ^2^	OR	95% CI	*p*-value
Non-PP patients (n = 68)	sTM (ng/mL)	Univariate analysis	0.027	0.008	13.004	1.028	1.013–1.043	<0.001*
		Multivariate analysis	0.014	0.011	1.592	1.014	0.992–1.037	0.207
	LUS	Univariate analysis	0.138	0.077	3.214	1.147	0.987–1.334	0.073
		Multivariate analysis	0.061	0.072	0.716	1.063	0.923–1.224	0.398
	Lactate (mmol/L)	Univariate analysis	0.217	0.077	7.872	1.242	1.068–1.445	0.005*
		Multivariate analysis	0.191	0.103	3.413	1.210	0.988–1.482	0.065
PP patients (n = 27)	sTM (ng/mL)	Univariate analysis	0.035	0.013	6.994	1.036	1.009–1.063	0.008*
		Multivariate analysis	0.004	0.024	0.029	1.004	0.959–1.051	0.864
	LUS	Univariate analysis	0.298	0.085	12.289	1.347	1.140–1.592	0.001*
		Multivariate analysis	0.340	0.163	4.369	1.405	0.926–1.847	0.127
	Lactate (mmol/L)	Univariate analysis	0.217	0.113	3.677	1.243	0.995–1.552	0.055
		Multivariate analysis	0.029	0.177	0.027	1.030	0.727–1.458	0.869

S.E., standard error; OR, odds ratio; CI, confidence interval; n, number; sTM, serum soluble thrombomodulin; LUS, lung ultrasound score. The symbol * indicates *p* < 0.05.

## 4 Discussion

Due to the highly heterogeneous nature of ARDS, developing reliable diagnostic tests and specific drug treatments is significantly challenging. To enhance the predictive accuracy for extrapulmonary ARDS, this study aims to establish a multiparameter predictive model. We selected sTM as a protein biomarker indicative of pulmonary endothelial injury and the LUS as a clinical diagnostic indicator of pulmonary pathophysiology. Additionally, we gathered a comprehensive set of clinical characteristics, including demographics, underlying causes of ARDS, comorbidities, physical findings, laboratory results, clinical scores, and therapeutic interventions. Using univariate Cox regression and Lasso regression analyses, we identified sTM and LUS as independent risk factors for ARDS mortality. Among the various clinical characteristics evaluated, only lactate levels were found to be an independent risk factor for ARDS mortality.

Thrombomodulin (TM) is an endothelial-specific transmembrane protein that can be cleaved into its soluble form (sTM) during endothelial activation or injury (Boehme et al., 1996). TM plays a critical role in the negative regulation of coagulation and inflammation (Ito and Maruyama, 2011). Given the abundant expression of sTM in the lungs and its detectability in serum (Kawanami et al., 2000), sTM serves as a biomarker for endothelial cell damage in the lungs. Elevated serum sTM levels indicate a reduction in the anticoagulant and anti-inflammatory capacities of pulmonary microvascular endothelial cells (Hofstra et al., 2008; Yang et al., 2014), which may exacerbate endothelial cell damage and create a vicious cycle. The potential of sTM as a serum biomarker for ARDS has garnered significant attention, with recent studies exploring its utility in predicting the onset and prognosis of ARDS (Orwoll et al., 2015; Sapru et al., 2015). Further research indicates that patients with extrapulmonary ARDS exhibit significantly elevated levels of sTM compared to those with pulmonary ARDS (Orwoll et al., 2015), suggesting that circulating sTM is more relevant for this subgroup rather than the predominantly pulmonary ARDS cases found in ICUs. Additionally, within the subcategories of extrapulmonary ARDS, those associated with non-pulmonary sepsis show markedly higher sTM levels compared to trauma-induced ARDS or ARDS of other etiologies (Garcia-Alvarez et al., 2014). This increase in sTM is also linked to higher mortality rates. Notably, although COVID-19-related ARDS is classified as pulmonary ARDS, it also presents with elevated serum sTM levels, with critically ill patients exhibiting significant and sustained increases (Sb et al., 2024). This indicates that endothelial injury is a prominent feature in the pathogenesis of severe COVID-19-related ARDS. These findings suggest that sTM, which reflects pulmonary endothelial activation, may serve as a valuable biomarker for identifying specific ARDS biophenotypes. This can aid in recognizing subgroups with similar pathophysiological characteristics. It is important to note that sTM is not routinely measured in clinical practice, and not all laboratories have the capability to quantify its levels.

Lung ultrasound has proven to be highly valuable in diagnosing ARDS. The global definition of ARDS incorporates ultrasound diagnostic criteria from the Kigali definition (Matthay et al., 2024). Study by Smit et al. (2023) has shown that the LUS method demonstrates greater accuracy in diagnosing and excluding ARDS compared to the modified Kigali method. LUS involves dividing both lungs into sections, with the commonly used method being the 12-zone technique. This approach assesses the extent of ventilation loss in both lungs by calculating the total score for all zones. Since lung heat dilution evaluates LUS in direct relation to extravascular lung water and quantitative computed tomography assesses LUS in direct relation to overall lung tissue density (Corradi et al., 2013), LUS serves as a semi-quantitative indicator of aeration status. An elevated LUS reflects alveolar ventilation loss and increased pulmonary shunting, the primary causes of severe hypoxemia in ARDS patients. LUS has been utilized in evaluating the diagnosis, treatment, and prognosis of ARDS patients (Pisani et al., 2019; Sayed et al., 2022; Smit et al., 2023; Huai and Ye, 2024). Due to its non-invasive and bedside nature, ultrasound presents unique advantages in resource-limited settings and during the implementation of prone positioning ventilation (Rousset et al., 2021; Mayo et al., 2022). However, comprehensive lung ultrasound training is essential before it can be effectively used as a predictive tool in clinical practice.

Lactate levels are often regarded as a reliable indicator of disease severity in critically ill patients (Zhang et al., 2022). Lactate is a metabolic byproduct of anaerobic glycolysis, and elevated lactate levels typically indicate increased production under hypoxic or ischemic conditions, reflecting a mismatch between production and clearance (Wardi et al., 2020). In the ICU, various types of shock often lead to elevated lactate levels due to reduced systemic tissue perfusion, an imbalance between oxygen supply and demand, and cellular hypoxia (Vincent et al., 2016). Additionally, in critically ill patients, elevated catecholamine levels and the production of multiple cytokines can promote glycolysis even under non-hypoxic conditions, resulting in hyperlactatemia (Haji-Michael et al., 1999). It is noteworthy that during ARDS, the lungs can release lactate at rates exceeding 60 mmol/h, far surpassing normal levels (Opdam and Bellomo, 2000; Iscra et al., 2002). This release is significantly correlated with the severity of lung injury, likely due to the promotion of glycolysis in lung tissues by inflammatory cells and cytokines (Brown et al., 1996; Haji-Michael et al., 1999). Therefore, when interpreting elevated serum lactate levels in the presence of systemic tissue hypoperfusion and ARDS, it is essential to consider the contribution of pulmonary lactate release alongside systemic hypoxia. Given that lactate levels are influenced by multiple factors, including hypoxia, tissue perfusion deficits, glucose metabolism disturbances, and liver and kidney function (Li et al., 2022), a single measurement of lactate concentration can be challenging (Weinberger et al., 2021). Currently, research on the prognostic value of lactate in ARDS patients is limited.

Using Lasso-Cox regression analysis, our study developed a nomogram based on sTM, LUS, and lactate levels. The analysis demonstrated that the nomogram incorporating these factors had higher predictive value for 28-day mortality in extrapulmonary ARDS patients compared to models using single indicators. Additionally, the nomogram outperformed traditional models such as APACHE II score and PaO_2_/FiO_2_ ratio. The combined use of sTM, LUS, and lactate provides a comprehensive assessment of pulmonary vascular endothelial damage, uneven dead space ventilation, pulmonary shunting, and the overall severity of the patient’s condition.

Several limitations of our study should be acknowledged. Firstly, the relatively small sample size is insufficient for robustly testing the performance of the predictive score and may have restricted the identification of other potential confounding factors that could impact the predictive value of our combined model for 28-day mortality. Secondly, the lack of external validation limits the applicability of our findings. Future studies should include external validation cohorts to verify the robustness of our predictive model. Thirdly, there may have been selection bias in identifying factors related to patient prognosis, which could have influenced the results.

## 5 Conclusion

In summary, we identified three significant factors distinguishing survivors from non-survivors among patients with extrapulmonary ARDS admitted to the ICU. We developed a composite nomogram aimed at predicting the 28-day mortality rate in patients with extrapulmonary ARDS. Further studies with larger sample sizes are required to validate these findings and refine the predictive model.

## Data Availability

The raw data supporting the conclusions of this article will be made available by the authors, without undue reservation.
